# Characterization of e-Government adoption in Europe

**DOI:** 10.1371/journal.pone.0231585

**Published:** 2020-04-17

**Authors:** Ainhoa Yera, Olatz Arbelaitz, Oier Jauregui, Javier Muguerza

**Affiliations:** Department of Computer Architecture and Technology, University of the Basque Country (UPV/EHU), Donostia-San Sebastián, Spain; National Institute of Public Finance and Policy, INDIA

## Abstract

The digital divide in Europe has not yet been bridged and thus more contributions towards understanding the factors affecting the different dimensions involved are required. This research offers some insights into the topic by analyzing the e-Government adoption or practical use of e-Government across Europe (26 EU countries). Based on the data provided by the statistical office of the European Union (Eurostat), we defined two indexes, the E-Government Use Index (EGUI) and an extreme version of it taking into account only null or complete use (EGUI^+^), and characterized the use/non use of e-Government tools using supervised learning procedures in a selection of countries with different e-Government adoption levels. These procedures achieved an average accuracy of 73% and determined the main factors related to the practical use of e-Government in each of the countries, e.g. the frequency of buying goods over the Internet or the education level. In addition, we compared the proposed indexes to other indexes measuring the level of e-readiness of a country such as the E-Government Development Index (EGDI) its Online Service Index (OSI) component, the Networked Readiness Index (NRI) and its Government usage component (GU). The ranking comparison found that EGUI^+^ is correlated with the four indexes mentioned at 0.05 significance level, as the majority of countries were ranked in similar positions. The outcomes contribute to gaining understanding about the factors influencing the use of e-Government in Europe and the different adoption levels.

## Introduction

Despite the efforts made by European governments and administrations in recent decades, the digital divide in the old continent still persists. The Digital Economy and Society Index (DESI) [1], a composite index which has been published annually since 2014 by the European Commission to measure the progress of the 28 European Union (EU) countries towards a digital economy and society, provides an idea of the digital divide in Europe. In particular, DESI regroups 34 indicators in five principal policy areas weighted as follows: 25% connectivity, 25% human capital, 15% use of Internet services, 20% integration of digital technology and 15% digital public services. From 2014 to 2019 the highest digital divide between the 28 EU countries (max-min) was increased 2%, from 32% to 34%. Analyzing the DESI of 2019, we observe that only one country achieved high DESI values (≥70%), whereas 61% of the countries (17) scored medium values (∈[50%, 70%)) and 36% of the countries (10) achieved low values (<50%). In contrast, in 2014 the great majority of the European countries (24/28) scored low DESI values and just 14% of them (4/28) showed medium values. Although the average value of DESI in Europe was improved from 39% in 2014 to 52% in 2019, it is clear that more forceful actions are called for bridging the digital divide.

One of the aspects affected by the consequences of the digital divide and also analyzed within DESI [1] is the e-Government use or adoption. E-Government has several aspects, including social, technical, economic, political, and public administrative but most works define the mission of e-Government as systems that use Information and Communication Technology (ICT) to provide citizens with a better service [2][3]. E-Government has been defined as the use of digital technology, specially Web-based applications, to enhance access to and efficiently deliver government information and services [4]. Although it has featured a substantial growth, development and diffusion, citizens in all developed and developing countries may not be willing to adopt such services [5].

According to Shareef et al. [2] e-Government has several aspects, including social, technical, economic, political, and public administrative. Most dominating concepts of e-Government arise from the technical perspective and a combination of the socio-economic and public administrative perspectives but in the academic literature, the adoption models offered so far are mainly conceptual. For instance, inside DESI e-Government is represented by the E-Government Development Index (EGDI) a composite index which has been published biannually in the UN e-Government Survey since 2010 [6] and which considers three aspects: telecommunications infrastructure, human capital and online services. In 2018 the average value of the EGDI for the 28 EU countries (80%) was rated as Very-High (>75%) and in line with DESI, the average EGDI score for the period 2010-2018 improved by 14% [6–10]. Although some digital divide related themes are still mentioned in the 2018 UN e-Government Survey [10], e.g. access, affordability, age, bandwidth, content, disability, education, gender, migration, location, mobile, speed and useful usage, it seems that the e-Government situation in Europe is promising, at least from a theoretical point of view.

Another conceptual analysis is provided by the World Economic Forum’s Networked Readiness Index (NRI), also referred to as Technology Readiness, which measures the propensity of countries to exploit the opportunities offered by Information and Communications Technology (ICT). It is published in collaboration with INSEAD (a graduate business school with campuses in Europe, Asia, and the Middle East), as part of their annual Global Information Technology Report (GITR) [11]. The report is regarded as the most authoritative and comprehensive assessment of how ICT impacts the competitiveness and well-being of nations. The index is a composite of three components: the environment for ICT offered by a given country or community (market, political, regulatory, and infrastructure environment), the readiness of the country’s key stakeholders (individuals, businesses, and governments) to use ICT and the usage of ICT among these stakeholders.

Beyond the conceptual models, some limited empirical studies exist. For instance, the study carried out by Schwester [12] in US municipalities, concludes that e-Government adoption is a function of financial, technical, and human resources. Holding all other factors constant, municipalities with higher operating budgets, more full-time IT staff, and technical resources are more likely to implement a comprehensive e-Government platform. However, extensive empirical studies among the actual users to validate and generalize the models are absent [2].

In this context, we consider the Eurostat Community Statistics on Information Society [13] (Eurostat CSIS Surveys) an opportunity to carry out an extensive empirical study in European countries. They stated that in 2019 the average e-Government use (EGU) in the 28 EU countries reached just 55%. The EGU is computed as the percentage of individuals who used the Internet to interact with public authorities, for example by obtaining information from public websites and downloading or submitting official forms.

The aim of this research is to offer some insights into the empirical e-Government adoption across Europe (26 EU countries). Inspired on the e-Government adoption options described by different authors [14][15][16] and based on the Eurostat CSIS Surveys’ [13] e-Government use (EGU) question, which asks about the contact of respondents with public authorities or public services, we defined two indexes: E-Government Use Index (EGUI) which is computed based on the four answers available for the e-Government use question, and EGUI^+^ an extreme version of the previous index computed based only on the two answers showing extreme levels of e-Government use (complete and null). With regard to EGUI^+^ we defined four levels of e-Government use (very high, high, low and very low) and characterized the extreme levels of e-Government use (null and complete) by applying supervised learning procedures to the Eurostat data of two countries from each of the four EGUI^+^ levels. To carry out this step, we built a total of eight datasets containing 14 independent variables selected from the Eurostat CSIS Surveys [13], and we built classification trees, classifiers with explaining capacities, based on the Consolidated Tree Construction [17] algorithm. These procedures achieved an average accuracy of 73% and determined the main factors related to the practical use of e-Government in each of the countries, e.g. the frequency of buying goods over the Internet or the education level.

In addition, we compared one of the indexes calculated using empirical data given by Eurostat (EGUI^+^) to the following composite indexes measuring the level of e-readiness of a country: E-Government Development Index (EGDI) and its Online Service Index (OSI) component provided by United Nations [6–9] and the Networked Readiness Index (NRI) and its Government usage component (GU) published by The World Economic Forum [11, 18–22]. The Significance Tests for Kendall’s Tau (T) [23] applied to the rankings provided by these four indexes indicated that all of them were correlated to the one given by the empirically calculated index EGUI^+^.

The paper proceeds with the description of the Eurostat data on e-Government practical use (EGU) and the indexes we defined to quantify this concept (EGUI, EGUI^+^) in Section Eurostat CSIS Surveys. In the next section, Characterization of extreme values of e-Government use (EGU), we show the results of the supervised learning procedures applied to EGU for a selection of countries. Section Comparison between EGUI^+^ and other indexes^+^ describes four indexes that measure the e-readiness of countries and features related to e-Government, EGDI and OSI [9]/ NRI and GU [11], and compares them with EGUI^+^ in terms of ranking differences on average for the 2009-2015 (annual indexes) / 2010-2016 (biannual indexes) periods in 26 EU countries. In Section Discussion theoretical and empirical studies on e-Government are argued. Finally, in Section Conclusions we present the main findings of the study.

## Eurostat CSIS Surveys

In this section we first describe the data on e-Government extracted from the Eurostat’s Community Statistics on Information Society (CSIS) 2009-2015 (Eurostat CSIS Surveys). Then we show the two indexes defined to characterize the e-Government adoption, EGUI and EGUI^+^, based on the information extracted from the Eurostat CSIS Surveys.

### Description of the Eurostat CSIS Surveys

From 2002 to the present, Eurostat CSIS Surveys have been annually conducted in all Member States, two countries of the European Free Trade Association (EFTA), candidate and accession countries to the EU. The data collection is based on Regulation (EC) 808/2004 [24] of the European Parliament and the Council of the European Union and since 2011 the transmission of microdata to Eurostat is mandatory.

The Eurostat CSIS Surveys collect data on access and use of information and communication technologies (ICT) from households and individuals. Their sampling frames are established by countries and respect the quality requirements for European statistics and are in line with legal requirements. The survey covers households with at least one member aged between 16 and 74 and individuals of an age between 16 and 74. Information on access to ICT, e.g. connection to the Internet, is collected at household level while statistics on the use of ICT, mainly on the use of the Internet, is gathered for individuals. According to the Eurostat’s Methodological Manual for the statistics on the Information Society (survey year 2015) [25] in general, one individual in the household will answer the household related questions having the household perspective in mind. This one individual can for instance be the head of the household or the individual which has been selected for the individual questions. The survey distinguishes between annual core subjects, which are included in the survey every year, and episodic topics on various ICT phenomena, which change in different survey years. In particular, there are six annual core subjects: access to ICT, use of computers, use of the Internet, e-Government, e-Commerce and e-Skills. In order to analyze variables of access and use of ICT in relation to household or individual characteristics, a number of background variables are collected. These include composition, income and regional location of the household as well as the age, gender, educational attainment and employment situation of individuals.

We were given access to the annual micro-datasets for the period 2009-2015, which we used for the analysis on the practical use of e-Government. Some questions in the micro-datasets varied from year to year, thus, for the analysis we used just the seven questions (q_i_) common to all the years and the eight background variables (b.v_i_), which are shown in Table 1 below.

**Table 1:** Questions (q_i_) and background variables (b.v_i_) selected from the CSIS Surveys for the characterization of the dependent variable (d.v_i_) e-Government practical use (EGU).

Code	Description	Type	Value*	Description	No. resp (%)	Mode
HH_CHILD	Number of children	b.v_i_	{0,1,2,3,4}	{0,1,2,3,≥4}	{64,20,13,1}	0
HH_IQ	Income quartile	b.v_i_	1	Lowest quartile	10	4
			2	Second lowest	18	
			3	Second highest	24	
			4	Highest quartile	31	
AGECLS	Age range	b.v_i_	1	≤15	1	4
			2	16-24	17	
			3	25-34	19	
			4	35-44	23	
			5	45-54	20	
			6	55-64	14	
			7	65-74	6	
			8	≥75	0	
SEX	Gender	b.v_i_	{1,2}	{Male,Female}	{49,51}	2
ISCED	Education level	b.v_i_	1	Primary/Lower secondary	21	2
			2	Upper secondary	49	
			3	Tertiary	30	
EMPST	Employment sit.	b.v_i_	1	Employee/self-employed	63	1
			2	Unemployed	8	
			3	Student	12	
			4	Not in the labor force	17	
OCC_ICT	ICT occupation	b.v_i_	{0,1}	{no,yes}	{60,3}	0
OCC_MAN	Manual occupation	b.v_i_	{0,1}	{no,yes}	{45,17}	0
IACC	Internet access	q_i_	{0,1}	{no,yes}	{7,93}	1
CU	Computer use	q_i_	1	> a year ago/never	1	3
			2	(3 months-a year) ago	3	
			3	< 3 months ago	96	
CFU	Computer freq. of use	q_i_	1	≤ once a month/year	5	3
			2	≤ once a week	15	
			3	(Almost) every day	76	
IU	Internet use	q_i_	1	> a year ago/never	0	3
			2	(3 months-a year) ago	3	
			3	< 3 months ago	97	
IFU	Internet freq. of use	q_i_	1	≤ once a month/year	6	3
			2	≤ once a week	16	
			3	(Almost) every day	75	
IBUY	Buy online	q_i_	1	> year ago/never	58	1
			2	(3 months-a year) ago	12	
			3	< 3 months ago	30	
**EGU**	**E-Government use**	**q_i_/d.v_i_**	**1**	**Null**	51	1
			**2**	OI**	16	
			**3**	OI** & DF**	9	
			**4**	OI** & DF** & SF**	24	

* 9 = no answer / don’t know,?/NA = no data available, and are not represented in the table.

**OI = obtain information, DF = download forms, SF = send filled forms.

Among the seven questions selected, one is at a household level and related to Internet access (IACC) whereas the remaining six are at individual level, two related to computer use (CU / CFU) and four related to the use of Internet (IU, IFU, IBUY and EGU). In the last row of Table 1 we show in bold the question selected as dependent variable (d.v_i_) to measure e-Government practical use, EGU, which was obtained by coding a question about the activities related to interaction with public services or administrations through the Internet for private purposes, providing four possible values: 1 if none of the three possible activities was carried out, 2 if the obtaining information activity (OI) was carried out, 3 if the OI and the downloading official forms (DF) activities were completed and 4 if OI, DF and sending filled in forms activities were carried out. The last column of Table 1 (Mode) provides the overall mode of the variables used. A country by country analysis revealed that the overall modes of nine of these variables shown in the table remained stable in all the countries: number of children (HH_CHILD), employment situation (EMPST), ICT occupation (OCC_ICT), manual occupation (OCC_MAN), Internet access (IACC), computer use (CU), computer frequency of use (CFU), Internet use (IU) and Internet frequency of use (IFU). Oppositely, six of these variables showed fluctuations among the countries analyzed in terms of mode values (see S1 Appendix): income quartile (HH_IQ), age range (AGECLS), gender (SEX), education level (ISCED), buy goods over the Internet (IBUY) and e-Government use (EGU). In these cases, although the mode was not the same for all the countries analyzed, we also found common values for a majority of them: e.g., in the case of highest income quartile (HH_IQ = 4) for 77% of the countries (20/26) and upper secondary education level (ISCED = 2) for 77% of the countries (20/26). So we could conclude from this analysis that the average social background characteristics of the respondents are similar for the 26 countries included in the analysis. In addition, the penultimate column in Table 1, No. resp (%), provides for each of the questions or variables, the percentage of respondents for each possible value/answer available, giving thus, an idea of the distribution of the sample.

Analyzing the micro-datasets, we realized that six countries were missing data for the year 2008 and thus, we focused our analysis on the period 2009-2015. United Kingdom and Croatia were removed from our analysis because they were missing data for two of the years (2009 and 2010) of the period of time of our scope. Therefore our analysis comprises a total of 767,691 surveys from 26 different EU countries. Table 2 illustrates the ample variability in the number of surveys for the countries selected over the years analyzed.

**Table 2:** Number of Eurostat CSIS Surveys analyzed for the period 2009-2015 in each country, S, average population, P, and percentage of surveys on average regarding the population, $\frac{S}{P}\left(\%\right)$.

		Number of Eurostat CSIS Surveys (S)									Pop. (P)	$\frac{S}{P}\left(\%\right)$
Country	Code	2009	2010	2011	2012	2013	2014	2015	Total	Avg	Avg	Avg
Austria	AT	4,634	4,620	3,178	3,454	3,371	3,291	3,455	26,003	3,715	8,430,714	0.04
Belgium	BE	4,049	4,109	3,872	3,899	4,000	3,794	0	23,723	3,389	11,032,857	0.03
Bulgaria	BG	2,832	3,325	4,876	4,064	4,682	5,167	4,847	29,793	4,256	7,331,143	0.06
Cyprus	CY	1,562	1,601	1,879	2,350	2,234	2,677	2,609	14,912	2,130	848,587	0.25
Czech Rep.	CZ	4,233	4,682	4,119	5,514	5,606	5,265	5,439	34,858	4,980	10,510,174	0.05
Denmark	DK	3,399	3,100	2,942	2,974	3,071	3,128	3,044	21,658	3,094	5,610,387	0.06
Estonia	EE	2,751	3,043	2,946	3,604	3,792	2,763	1,919	20,818	2,974	1,322,139	0.22
Greece	EL	1,538	1,568	1,865	1,482	1,813	2,080	2,522	12,868	1,838	10,985,896	0.02
Spain	ES	8,586	9,268	9,295	8,312	8,509	8,837	9,076	61,883	8,840	46,585,966	0.02
Finland	FI	1,989	2,053	2,164	2,141	2,107	1,967	2,072	14,493	2,070	5,423,568	0.04
France	FR	2,180	3,323	4,819	6,517	5,675	4,831	6,711	34,056	4,865	65,699,856	0.01
Hungary	HU	4,092	4,373	4,793	4,811	4,656	4,844	4,593	32,162	4,595	9,914,884	0.05
Ireland	IE	4,321	4,520	3,683	6,653	6,815	6,054	5,401	37,447	5,350	4,623,020	0.12
Italy	IT	18,133	18,461	19,143	18,611	19,229	19,539	20,582	133,698	19,100	59,982,585	0.03
Lithuania	LT	6,551	6,484	6,150	5,931	5,947	6,450	4,262	41,775	5,968	2,989,057	0.20
Luxembourg	LU	1,126	1,204	1,060	1,297	1,134	1,072	1,132	8,025	1,146	537,812	0.21
Latvia	LV	0	4,252	4,742	4,043	4,264	3,533	4,306	25,140	3,591	2,031,467	0.18
Malta	MT	583	634	812	709	852	881	856	5,327	761	426,943	0.18
Netherlands	NL	3,304	3,323	3,392	3,563	3,459	2,954	3,435	23,430	3,347	16,778,549	0.02
Norway	NO	878	803	856	778	842	854	902	5,913	845	5,042,976	0.02
Poland	PL	5,746	6,568	6,341	6,080	5,285	10,642	4,844	45,506	6,501	38,028,942	0.02
Portugal	PT	2,578	2,745	2,799	3,126	3,415	3,689	3,992	22,344	3,192	10,474,191	0.03
Romania	RO	4,731	5,688	6,154	6,216	7,819	8,570	9,405	48,583	6,940	20,026,908	0.03
Sweden	SE	3,207	2,976	2,124	1,033	1,110	1,067	966	12,483	1,783	9,576,891	0.02
Slovenia	SI	1,136	1,213	1,235	1,210	1,384	1,318	1,157	8,653	1,236	2,057,090	0.06
Slovakia	SK	2,682	3,025	2,930	3,357	3,593	3,320	3,233	22,140	3,163	5,408,795	0.06

As shown in the table, in the years analyzed on average, the number of CSIS Surveys is not proportional to the population of the countries (see columns 11 and 12 respectively): e.g. the biggest number of surveys which corresponds to Italy (19,100) is 25 times higher than the smallest number which corresponds to Malta (761), whereas the population of this second country (426,943) is 140 times lower than that of the first one (59,982,585). However, although the percentage of number of CSIS Surveys for the 26 countries regarding the population changes (see last column of Table 2), in every case, the number of responses used ensures a confidence level above 95% and an error margin below 5%. This would always be a pessimistic estimation since the target population of CSIS Surveys is in the age range 16-74 and thus, it is always smaller than the population shown in Table 2. In general, in countries with small populations, e.g., Malta and Luxembourg, this percentage was higher than in countries with big populations, e.g., France and Italy, (0.20% > 0.02% on average).

According to the literature review the information provided in the surveys seems to be promising for the empirical study proposed in this contribution. In the study conducted by Carter and Bélanger [5], perceived ease of use, compatibility and trustworthiness appear to be significant predictors of citizens’ intention to use an e-Government service. In an empirical study conducted by Shareef et al. [2], the authors observed that e-Government adoption behavior differs based on service maturity levels, i.e., when functional characteristics of organizational, technological, economical, and social perspectives of e-Government differ. A user will not arrive at an intention to use an e-Government system, which requires computer knowledge to get a competitive advantage, unless the user has competence from experience in the use of modern ICT. From technological, behavioral, economic, and organizational perspectives, it is anticipated that failing to get hands-on experience of technology will not create in the user an attitude favorable to adopting the system. Therefore, from organizational perspectives, computer self-efficacy is an important predictor of whether a user will adopt an e-Government system instead of using traditional government services. Bélanger and Carter [14] propose a model of e-Government trust composed of disposition to trust, trust of the Internet (TOI), trust of the government (TOG) and perceived risk. Results from a citizen survey (214 responses) indicate that disposition to trust positively affects TOI and TOG, which in turn affects intentions to use an e-Government service. According to Nam [15] the degree of e-Government use for a specific purpose is predicted by five sets of determinants: psychological factors of technology adoption, civic mindedness, information channels, trust in government, and socio-demographic and personal characteristics. Socio-demographic conditions influence usage level of various transactional services provided by e-Government. Perceived ease of use facilitates the acquisition of general information through e-Government.

### E-Government use indexes: EGUI / EGUI^+^

The literature identifies different e-Government adoption levels. Bélanger et al. [14] differentiated the dependent variable “Adoption” into two sub-groups:

- Adoption 1: Decision to accept and use an e-Government system to view, collect information, and/or download forms for different government services as the user requires with the positive perception of receiving a competitive advantage. This would include the situations with information transference from the government to the user but not in the other sense. This adoption level is represented in Table 1 by the following answers available for the e-Government use question (EGU): EGU_2_ = obtain information (OI), and EGU_3_ = obtain information (OI) and download forms (DF).
- Adoption 2: Decision to accept and use an e-Government system to interact with, and seek government services, and/or search for queries for different government services as the user requires with the positive perception of receiving a competitive advantage. In this case, the user engagement is bigger and the communication done in the user to government sense, is also done using electronic facilities. This adoption level is represented in Table 1 by the answer to the e-Government use question (EGU) which includes the send filled forms action (SF): EGU_4_.

On the other hand, Nam [15] and Thompson et al. [16] identified three main purposes of e-Government use: information use, service use or engaging in electronic transactions with government and policy research or to participate in government decision making. The first two, could be equivalent to the Adoption 1 and 2 defined in [14].

Bearing these definitions in mind, in order to quantify e-Government adoption we defined two indexes, EGUI and EGUI^+^, which are computed as ratios between the number of answers (#) to the question on EGU (EGU_i_) that reveal some level of e-Government use (i ∈ {2,3,4}) and the ones that indicate no use (i = 1). Eq 1 specifies how the two defined e-Government Use Indexes are computed. As it can be observed, EGUI takes into account the Adoption 1 or information use idea and EGUI^+^ is an extreme version of EGUI that only involves the users engaged in electronic transactions, null use against complete use (#EGU_i_, i ∈ {1,4}).

$$\begin{array}{c} EGUI=\frac{\sum_{i=2}^{4}\#EGU_{i}}{\#EGU_{1}}\;;\;EGUI+=\frac{\#EGU_{4}}{\#EGU_{1}} \end{array}$$ (1)

In Table 3 we provide the list of countries ordered according to the EGUI^+^ ranking, the total number of possible answers gathered for the EGU question (#EGU_i_, i ∈{1, 2, 3, 4}) and the EGUI and EGUI^+^ values.

**Table 3:** Average values of EGUI and EGUI^+^ (2009-2015) in the 26 EU countries analyzed.

	#EGU_i_				Value		
Country	i = 1	i = 2	i = 3	i = 4	EGUI	EGUI^+^	EGUI^+^ level
DK	2,955	3,718	1,701	13,284	6.3	4.5	Very high
NO	1,263	1,003	771	2,876	3.7	2.3	Very high
FI	3,564	2,656	1,656	6,617	3.1	1.9	High
NL	7,231	3,986	1,278	10,935	2.2	1.5	High
SE	3,285	2,573	2,085	4,540	2.8	1.4	High
FR	11,844	5,263	4,497	12,452	1.9	1.1	High
IE	16,406	2,677	1,647	16,717	1.3	1.0	High
EE	8,202	4,251	394	7,971	1.5	1.0	High
AT	9,747	4,993	4,451	6,812	1.7	0.7	Low
LU	2,948	1,083	1,982	2,012	1.7	0.7	Low
ES	26,320	11,815	7,409	16,339	1.4	0.6	Low
PT	11,689	2,784	921	6,950	0.9	0.6	Low
SI	3,222	1,690	1,896	1,845	1.7	0.6	Low
HU	14,802	5,803	3,127	8,430	1.2	0.6	Low
LT	23,661	4,532	329	13,253	0.8	0.6	Low
LV	10,589	7,706	1,263	5,582	1.4	0.5	Low
BE	11,525	4,528	2,303	5,367	1.1	0.5	Low
MT	2,636	723	776	1,192	1.0	0.5	Low
EL	6,375	2,842	949	2,702	1.0	0.4	Very low
CY	7,678	1,799	2,247	3,188	0.9	0.4	Very low
SK	10,210	5,250	2,769	3,911	1.2	0.4	Very low
IT	88,551	13,377	13,555	18,215	0.5	0.2	Very low
BG	18,996	5,231	1,809	3,757	0.6	0.2	Very low
CZ	21,731	6,969	2,169	3,989	0.6	0.2	Very low
PL	29,955	6,473	3,708	5,370	0.5	0.2	Very low
RO	39,003	5,751	1,268	2,561	0.2	0.1	Very low

Based on the EGUI^+^ values shown in Table 3 we were able to rate the countries into four different e-Government use levels: very high (≥2.0), high (∈[1.0, 2.0)), low (∈[0.5, 1.0)) and very low (<0.5). As a result, two countries were rated as having a very high level (DK, NO), six as having a high level (FI, NL, SE, FR, IE, EE), 10 with a low level (AT, LU, ES, PT, SI, HU, LT, LV, BE, MT) and eight with a very low level (EL, CY, SK, IT, BG, CZ, PL, RO).

## Characterization of extreme values of e-Government use (EGU)

Aiming to obtain a greater understanding of e-Government adoption, we characterized the factors involved in the EGUI^+^ index. As a preliminary study we computed the Pearson correlation for the 26 countries to get the correlation of the 14 independent variables with the two extreme values of the dependent variable e-Government use, (EGU): EGU_1_ (null) and EGU_4_ (complete). This provided us with a global picture of the factors which most influenced the extreme values of e-Government use in Europe. To facilitate the interpretation of the correlation results, the irrelevant answers (9 = no answer / don’t know) were removed for this analysis.

According to Pearson, a high frequency to buy goods over the Internet (IBUY) was the variable with the highest correlation coefficient (|*r*| = 0.43) with e-Government use (EGU), which according to Cohen [26] suggests a medium strength correlation (0.3 < |*r*|<0.5). In addition, a medium strength correlation (|*r*| = 0.34) was also found between education level (ISCED) and e-Government use. Finally, manual occupation (OCC_MAN) and Internet frequency of use (IFU) were found to be inversely and positively correlated with EGU respectively (|*r*| = 0.27), which are considered nearly medium strength correlations. In all the cases the p-value of the test was lower than the significance level alpha, 0.05 and thus, the correlations found are significant although the majority of the values are of small strength (0.1 < |*r*|<0.3).

To find more specific characteristics of e-Government use, we used the supervised learning algorithm Consolidated Tree Construction (CTC) [17], which beyond a specific discriminating capacity to distinguish between the two extreme levels of e-Government use (null and complete), provided a particular and stable description of the most influential variables for each level. For the analysis we selected two countries from each of the four EGUI^+^ levels defined, very high, high, low and very low.

In particular an experiment was run in Weka [27] with CTC for the eight countries selected, using the 14 independent variables and the dependent variable EGU with two possible values, null (EGU_1_) and complete (EGU_4_). A ten-fold cross-validation (10-fold CV) strategy was used for validation. Table 4 shows the characteristics of the datasets and the obtained classification rates. As can be observed the datasets are quite unbalanced in the majority of countries selected: columns #EGU_i_ and #EGU_i_ (%). Thus, in order to obtain a better characterization of the minority EGU class in each country, CTC was run using a distribution of the minority class of 50% and 2% of each dataset as the minimum number of instances per leaf, which limits the minimum size of any decision node to the specified value.

**Table 4:** Number of answers for the extreme levels of e-Government use (#EGU_1_, i ∈{1,4}) and CTC average results in eight countries with four EGUI^+^ levels.

		#EGU_i_		#EGU_i_ (%)		CTC average results			
Country	EGUI^+^ level	i = 1	i = 4	i = 1	i = 4	Pr	Re	Fm	Acc
DK	Very high	2,955	13,284	18	82	0.85	0.83	0.84	0.83
NO	Very high	1,263	2,876	31	69	0.76	0.74	0.74	0.74
IE	High	16,406	16,717	50	50	0.73	0.73	0.73	0.73
EE	High	8,202	7,971	51	49	0.73	0.73	0.73	0.73
LV	Low	10,589	5,582	65	35	0.77	0.74	0.74	0.73
BE	Low	11,525	5,367	68	32	0.77	0.71	0.72	0.71
PL	Very low	29,955	5,370	85	15	0.86	0.74	0.77	0.74
RO	Very low	39,003	2,561	94	6	0.94	0.67	0.75	0.67

According to Table 4 the average results achieved by the CTC trees in terms of Precision (Pr), Recall (Re), F-measure (Fm) and Accuracy (Acc) were good with values over 0.71 in the three groups, except in Romania where Recall and Accuracy scored 0.67. This is not surprising since Romania has a very unbalanced dataset, with 94% of the surveys being of null e-Government use type (#EGU_1_), which reduces the Recall and Accuracy of the minority class.

**Fig 1:**
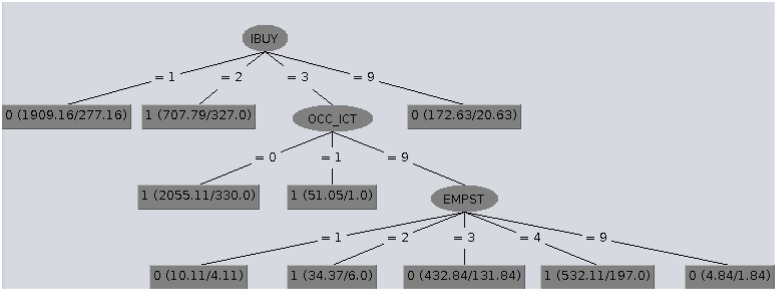
CTC tree obtained for Denmark which has a very high EGUI^+^ level.

**Fig 2:**
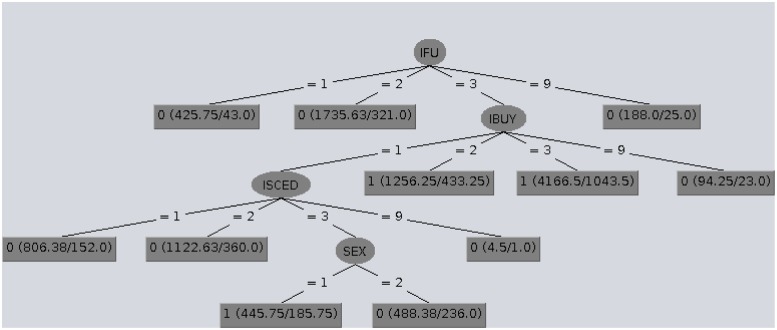
CTC tree obtained for Belgium which has a low EGUI^+^ level.

**Fig 3:**
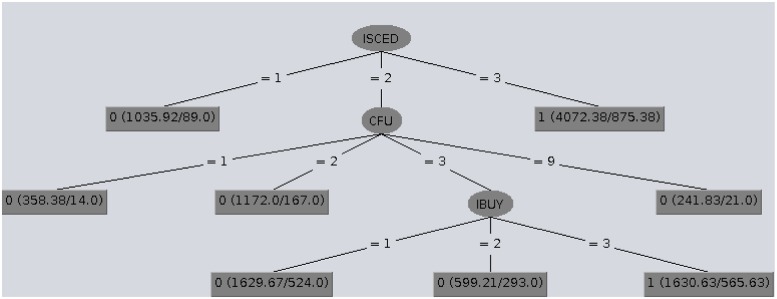
CTC tree obtained for Poland which has a very low EGUI^+^ level.

The structures of the classification trees provide an explanation of the classification. In Figs 1, 2 and 3 CTC trees obtained for Denmark, Belgium and Poland are shown by way of example. In the CTC trees displayed, in the leaf nodes the first number (0/1) represents the class given to the leaf node, the null or complete e-Government use level (EGU_1_/EGU_4_), whereas inside the parenthesis, the values before and after the slash represent the number of examples involved and the number of misclassified examples respectively.

Globally analyzing these structures, we concluded that excluding the countries with a very low EGUI^+^ level, complete e-Government use (EGU_4_) was closely related to recent online shopping (IBUY = 3), whereas the same action carried out more long time ago (IBUY = 1) seemed to be connected to null e-Government use (EGU_1_). Table 5 summarizes the main rules provided by the CTC trees for each country and their descriptions are given in sections Countries with a very high EGUI^+^ level: Denmark and Norway, Countries with a high EGUI^+^ level: Ireland and Estonia, Countries with a low EGUI^+^ level: Latvia and Belgium and Countries with a low EGUI^+^ level: and Countries with a very low EGUI^+^ Poland and Romania.

**Table 5:** Main rules provided by the CTC trees for complete and null levels of e-Government use (EGU).

	Main rules for extreme levels of e-Government use (EGU).	
Country	Null: EGU_1_	Complete: EGU_4_
DK	IBUY = 1	IBUY = 3 & OCC_ICT = 0
	IBUY = 3 & EMPST = 1/3	IBUY = 2
NO	IFU = 3 & IBUY = 1	IFU = 3 & IBUY = 3
	IFU≠3	IFU = 3 & IBUY = 2 & ISCED≠1
IE	IBUY = 1 & CFU≠3	IBUY = 3 & IFU = 3 & ISCED = 3
	IBUY = 3 & IFU = 3 & ISCED = 2	IBUY = 1 & CFU = 3 & ISCED≠3 & AGECLS≠2
EE	IBUY = 1 & OCC_MAN≠0	IBUY = 3 & ISCED≠1
	IBUY = 1 & OCC_MAN = 0 & CFU≠3	IBUY = 1 & OCC_MAN = 0 & CFU = 3
LV	ISCED = 1	ISCED = 3 & EMPST≠4
	ISCED = 2 & IBUY = 1	ISCED = 2 & IBUY≠1 IFU≠3
BE	IFU = 3 & IBUY = 1 &	IFU = 3 & IBUY≠1
	ISCED≠3	
PL	ISCED = 1	ISCED = 3
	ISCED = 2 & CFU≠3	ISCED = 2 & CFU = 3 & IBUY = 3
RO	OCC_MAN≠0	OCC_MAN = 0

Analyzing Table 5, the following variables were found to have a close connection with extreme e-Government use levels (null and complete), listed in a descending number of appearances in the main 29 rules presented for the eight countries: Buy goods over the Internet (IBUY) = 21/29, Education level (ISCED) = 14/29, Internet frequency of use (IFU) = 8/29, Computer frequency of use (CFU) = 6/29, Manual occupation (OCC_MAN) = 5/29, Employment situation (EMPST) = 2/29 and ICT occupation (OCC_ICT) = 1/29.

### Countries with a very high EGUI^+^ level: Denmark and Norway

In Denmark the citizens who rarely bought goods over the Internet (IBUY = 1) and those who had done online shopping recently and were employees/self-employees or students (IBUY = 3 & EMPST = 1/3) did not use e-Government tools (EGU_1_). On the other hand, Danish citizens who did use e-Government tools (EGU_4_) had bought online quite recently and were not ICT professionals (IBUY = 3 & OCC_ICT = 0) or had done online shopping between three months and a year previously (IBUY = 2). These main CTC rules found for EGU in Denmark are also available in the most representative nodes of each class (0:EGU_1_/1:EGU_4_) in Fig 1.

In Norway the people who used the Internet almost everyday but had not bought goods over the Internet for a time (IFU = 3 & IBUY = 1) and the ones who did not daily use the Internet (IFU≠3) did not use e-Government tools. Conversely, Norwegian people who used the Internet almost everyday and had recently bought goods over the Internet (IFU = 3 & IBUY = 3) did use e-Government. In addition, Norwegians who used the Internet almost daily, had bought goods over the Internet quite recently and did not have a low education level (IFU = 3 & IBUY = 2 & ISCED≠1) also used e-Government tools.

### Countries with a high EGUI^+^ level: Ireland and Estonia

In Ireland the citizens not using e-Government tools (EGU_1_) were those who hardly ever bought goods over the Internet and who did not use the computer daily (IBUY = 1 & CFU≠3) together with those who hardly ever did online shopping, used the computer almost daily but did not have a high education level (IBUY = 1 & CFU = 3 & ISCED≠3). On the other hand, the Irish citizens who recorded a complete use of e-Government (EGU_4_) were those who had bought goods over the Internet recently, used the Internet daily and had a high education level (IBUY = 3 & IFU = 3 & ISCED = 3). Additionally, Irish people who had shopped online recently, used the Internet almost daily, had a medium education level and were not in the age range of 16-24 years also used e-Government tools (IBUY = 3 & IFU = 3 & ISCED = 2 & AGECLS≠2).

In Estonia citizens who had not bought online for long time and had a manual occupation (IBUY = 1 & OCC_MAN≠0), as well as those who had not shopped online for a long time, but did not have a manual occupation and did not use the computer daily (IBUY = 1 & OCC_MAN = 0 & CFU≠3) did not use e-Government tools. However, Estonian citizens who had bought goods over the Internet recently and did not have a low education level (IBUY = 3 & ISCED≠1) or those who had bought online long time ago, did not have a manual occupation but used the computer almost every day (IBUY = 1 & OCC_MAN = 0 & CFU = 3), did use e-Government tools.

### Countries with a low EGUI^+^ level: Latvia and Belgium

In Latvia, people with a low education level (ISCED = 1) and those with a medium education level who had bought goods over the Internet a long time ago (ISCED = 2 & IBUY = 1) had no inclination to use the e-Government tools (EGU_1_). On the other hand, Latvians with a high education level who were not retired (ISCED = 3 & EMPST ≠ 4) and those with a medium education level who had bought online in the previous 12 months (ISCED = 2 & IBUY≠1) did use such tools (EGU_4_).

In Belgium the null use of e-Government is related to citizens who did not use the Internet daily (IFU≠3) along with the ones who used it almost daily but had not bought goods over the Internet for a long time and did not have a high education level (IFU = 3 & IBUY = 1 & ISCED≠3). On the other hand, Belgian citizens making complete use of e-Government, used the Internet daily and had shopped online within the previous 12 months (IFU = 3 & IBUY≠1). The most representative nodes of each class (0:EGU_1_/1:EGU_4_) in the CTC tree shown in Fig 2 also exhibit the main CTC rules found for EGU in Belgium.

### Countries with a very low EGUI^+^ level: Poland and Romania

In Poland citizens with a low education level (ISCED = 1) together with those with a medium education level who did not use the computer daily (ISCED = 2 & CFU≠3) showed a null trend towards the use of e-Government (EGU_1_). On the other hand, Poles who used e-Government tools (EGU_4_) had a high (ISCED = 3) or a medium education level, used the computer almost daily and had bought goods over the Internet quite recently (ISCED = 2 & CFU = 3 & IBUY = 3). The most representative nodes of each class (0:EGU_1_/1:EGU_4_) shown in Fig 3 also illustrate the main CTC rules found for EGU in Poland.

Romania was the only country where the two extreme e-Government use levels, null (EGU_1_) and complete (EGU_4_), were characterized by two rules that involved a single factor, manual occupation (OCC_MAN): manual workers (OCC_MAN≠0) did not use the e-Government tools, whereas non manual workers did use them.

## Comparison between EGUI^+^ and other indexes

Aiming to analyze if the performance of the index we defined to measure the practical e-Government use is similar to other conceptual indexes broadly used as indicators of related features such as e-readiness of a country, we selected four indexes and compared them to EGUI^+^: E-Government Development Index (EGDI) and its Online Service Index (OSI) component, and the Networked Readiness Index (NRI) and its Government usage (GU) component. Next we describe the indexes mentioned and the comparison carried out.

### Description of the indexes

From 2001 to the present, The United Nations Department of Economic and Social Affairs (UNDESA) has published the UN E-Government Survey [10]. In 2003 this survey began to provide an analysis of the progress in using e-Government via the E-Government Development Index (EGDI), a composite index based on the weighted average of three normalized (norm.) indices, assigning one third of weight to each of them (see Eq 2): the Telecommunications Infrastructure Index (TII), the Human Capital Index (HCI) and the Online Service Index (OSI). As a composite indicator, the EGDI is used to measure the readiness and capacity of national institutions to use ICTs to deliver public services [10]. Prior to the normalization of the three component indicators, the Z-score standardization procedure is implemented for each component indicator to ensure that the overall EGDI is decided equally by the three component indexes.

$$\begin{array}{c} EGDI=\frac{1}{3}\left(TII_{norm.}+HCI_{norm.}+OSI_{norm.}\right) \end{array}$$ (2)

The TII index is based on data provided by the International Telecommunications Union (ITU) and is computed as the weighted average of the Z-score of five component indicators: Internet users, Fixed telephone subscribers, Mobile/Cellular telephone subscription, Active mobile broadband subscription and Fixed broadband. [10]

The HCI index is based on data supplied by the United Nations Educational, Scientific and Cultural Organization (UNESCO), and it is computed as the weighted arithmetic mean with one-third weight assigned to the adult literacy rate and two-ninths weight assigned to the gross enrollment ratio, estimated years of schooling and mean years of schooling. [10]

The OSI index, one of the three component indicators of the EGDI index described in Eq 2 that was selected for the analysis, is a composite normalized score based on an independent survey questionnaire, conducted by UNDESA, which assesses the national online presence of all 193 United Nations Member States. The survey questionnaire computes several features related to online service delivery, including whole-of-government approaches, open government data, e-participation, multi-channel service delivery, mobile services, usage up-take, digital divide as well as innovative partnerships through the use of ICTs. The majority of questions are of binary type (yes, no) and the total number of points scored by a country is normalized to a range of 0 to 1. The value for a given country is computed as the subtraction between the total number of points scored by that country and the lowest score for any country divided by the range of values for all countries in the survey; for instance, let 114 be the score of a country *“x”*, 0 the lowest score of any country and 153 the highest one, then the Online Service Index of *“x”* will be $0.7451=\frac{\left(114-0\right)}{\left(153-0\right)}$. [10]

The World Economic Forum has been annually publishing The Global Information Technology Report [11] since 2001, where the Networked Readiness Index (NRI) is provided. As shown in Eq 3, the NRI is a composite index computed as the weighted average of four main subindexes, being all the weights a quarter: Environment subindex, Readiness subindex, Usage subindex and Impact subindex.

$$\begin{array}{c} NRI=\frac{1}{4}\left(Environment_{subindex}+Readiness_{subindex}+Usage_{subindex}+Impact_{subindex}\right) \end{array}$$ (3)

The Environment subindex assesses the extent to which a country’s market conditions and regulatory framework support entrepreneurship, innovation, and ICT development [11]. This subindex is computed as the weighted average of two pillars (using weights of one half) which are based on nine indicators respectively: the Political and regulatory environment pillar (e.g., intellectual property rights protection and prevalence of software piracy) and the Business and innovation environment one (e.g., red tape and ease of starting a business).

The Readiness subindex measures the extent to which a country has in place the infrastructure and other factors to support the uptake of ICTs [11]. This subindex is computed as the weighted average of four pillars (using weights of one quarter). The Infrastructure pillar is based on four indicators, e.g., mobile network coverage, international Internet bandwidth, secure Internet servers etc. The Affordability pillar is based on three indicators, e.g., mobile telephony usage costs and broadband Internet subscription costs. The Skills pillar is based on four indicators, e.g., enrollment rate in secondary education and the overall quality of the education system.

The Usage subindex of NRI assesses the level of ICT adoption by a society’s main stakeholders: government, businesses and individuals [11]. In particular, the Usage subindex is computed as the weighted average of three pillars (using weights of one third). The Individual usage pillar is based on on seven indicators (e.g., mobile telephony penetration and Internet usage). The Business usage pillar is based on six indicators (e.g. firm-level technology absorption and capacity for innovation). The Government usage pillar (GU) used in this analysis, assesses the leadership and success of the government in developing and implementing strategies for ICT development, as well as in using ICTs, as measured by the availability and quality of government online services [11]. The Government usage pillar is computed as the average of three indicators: the importance of ICTs to government vision, the Government Online Service Index and the Government success in ICT promotion.

Finally, the Impact subindex measures the broad economic and social impacts accruing from ICTs [11]. This subindex is computed as the weighted average of two pillars (using weights of one half) which are based on four indicators respectively: the Economic impacts pillar (e.g., number of patent applications and impact of ICTs on business models) and the Social Impacts pillar (e.g., impact of ICTs on access to basic services and Internet access in schools).

In particular, about half of the 53 individual indicators used to compute the NRI are sourced from international organizations, mainly from the International Telecommunication Union (ITU), the World Bank, the United Nations Educational, Scientific and Cultural Organization (UNESCO) and other UN agencies [11]. The other half of the NRI indicators are derived from the World Economic Forum’s annual Survey which is administered annually to over 14,000 business executives in all the economies included in the NRI [28]. The Survey represents a unique source of insight into many critical aspects related to a country’s enabling environment, the preparedness of its population, ICT usage, and ICT impacts. When a country misses five or 10 percent of all indicators involved, its NRI is not computed.

In summary, three of the conceptual indexes/pillars used in the comparison, EGDI, NRI and its GU pillar, as well as the indexes we define are composite indexes computed based on a frequently used approach named equal-weight [29]. Although the number of indicators used in each of them is different, the indexes compared are among the most cited ones [30][29]. To this regard, a critical review on the concept of e-readiness made by Danish Dada [31] pointed out that indexes measuring e-readiness do not completely reflect the possibility of achieving development from ICTs in developing countries and suggests to consider the level of the individuals within the organization using the technology in order to obtain a more accurate measure. In this regard, the comparison shown in next sections contributes to enrich the idea of e-readiness provided by the four conceptual indexes selected, by adding information on the real use of e-Government services.

### Ranking comparison

In order to study the relationship between the practical use of e-Government (EGUI^+^) and the level of e-Government readiness (EGDI), network readiness (NRI), national online presence (OSI) and ICT adoption by government (GU), we present the values for these indexes (see Table 6) and we compared their rankings for the 26 countries analyzed. For the comparison we tried to use similar time periods, 2009-2015 period for the annual indexes or indicators, EGUI^+^, NRI and GU [11, 18–22], and 2010-2016 period for the biannual indexes or indicators, EGDI and OSI [6–9].

**Table 6:** EGUI^+^, EGDI, OSI, NRI and GU ranking comparison for the 26 countries analyzed.

Index values						Ranking (R) differences with EGUI^+^: *R*_*X*_ − *R*_*EGUI*^+^_							
Co.	EGUI^+^	EGDI	OSI	NRI	GU	Co.	EGDI	Co.	OSI	Co.	NRI	Co	GU
DK	4.50	0.84	0.74	5.57	5.21	**IT**	**-8**	**IT**	**-10**	**BE**	**-7**	**MT**	**-12**
NO	2.28	0.83	0.79	5.52	5.20	**BE**	**-7**	**ES**	**-8**	**CY**	**-6**	**EE**	**-7**
FI	1.86	0.82	0.77	5.75	5.03	ES	-4	**BE**	**-6**	**CZ**	**-6**	BE	-4
NL	1.51	0.87	0.87	5.57	5.08	FR	-4	FR	5	**MT**	**-6**	CZ	-4
SE	1.38	0.83	0.74	5.82	5.32	PL	-4	LT	-5	LU	-4	RO	-4
FR	1.05	0.84	0.88	5.08	5.04	NL	-3	PL	-5	SE	-4	CY	-3
IE	1.02	0.74	0.61	4.99	4.44	CZ	-2	EE	-2	PL	-3	SE	-3
EE	0.97	0.79	0.75	5.08	5.36	LT	-2	NL	-2	AT	-2	BG	-2
AT	0.70	0.77	0.72	5.17	4.71	MT	-2	RO	-2	FI	-1	PT	-2
LU	0.68	0.75	0.60	5.29	4.84	AT	0	CZ	-1	IT	-1	LT	-1
ES	0.62	0.80	0.85	4.52	4.59	EE	0	AT	0	NL	-1	LU	-1
PT	0.59	0.67	0.61	4.64	4.75	EL	0	MT	1	RO	-1	ES	1
SI	0.57	0.70	0.58	4.55	4.15	RO	0	CY	2	EE	0	FR	1
HU	0.57	0.67	0.60	4.22	3.89	SE	0	EL	2	LT	1	IT	1
LT	0.56	0.72	0.69	4.54	4.48	LU	1	FI	2	PT	1	NL	1
LV	0.53	0.66	0.58	4.29	3.79	BG	2	HU	2	SI	2	PL	1
BE	0.47	0.76	0.66	5.04	4.48	DK	2	LV	2	SK	2	AT	2
MT	0.45	0.68	0.57	4.85	5.06	NO	2	NO	2	BG	3	DK	2
EL	0.42	0.67	0.53	3.94	3.46	SI	2	PT	2	DK	3	NO	2
CY	0.42	0.60	0.49	4.57	4.10	CY	3	BG	3	FR	3	LV	3
SK	0.38	0.60	0.44	4.01	3.38	FI	3	SE	3	LV	3	SI	3
IT	0.21	0.71	0.62	4.14	3.47	SK	3	SI	4	NO	3	HU	4
BG	0.20	0.59	0.43	3.85	3.54	HU	4	SK	4	IE	4	SK	4
CZ	0.18	0.63	0.46	4.41	3.60	LV	4	LU	5	EL	5	EL	5
PL	0.18	0.64	0.54	4.05	3.28	IE	5	*DK*	*6*	*ES*	*6*	FI	5
RO	0.07	0.57	0.46	3.92	3.52	PT	5	*IE*	*6*	*HU*	*6*	*IE*	*8*

Specifically we computed the number of positions won or lost (positive or negative value) by the countries from the EGDI, OSI, NRI and GU rankings to the EGUI^+^ ranking, which in general terms is low (see Table 6). As shown in Table 6, we grouped the countries into three different sets using ±5 positions as a threshold for the ranking differences appreciated (nearly 20% of the ranking) represented by the following codes: blue-bold if they drop more than five positions, green-roman if they drop or gain fewer than five positions (stable countries) and red-italic if they gain more than five positions.

According to Table 6, for a great majority of the countries involved in the analysis, 84.6% on average (green-roman ones), the practical e-Government adoption does match the features measured by the conceptual indexes, EGDI, OSI, NRI and GU. To this regard, we found sixteen countries (62%) appearing in all the groups with small ranking differences (stable countries): Austria (AT), Bulgaria (BG), Greece (EL), Finland (FI), France (FR), Lithuania (LT), Luxembourg (LU), Latvia (LV), Netherlands (NL), Norway (NO), Poland (PL), Portugal (PT), Romania (RO), Sweden (SE), Slovenia (SI) and Slovakia (SK). In addition, we observed that EGDI is the most similar index to EGUI^+^, since 92% of the countries (24/26) are of stable type. Thus, in countries where the quality of e-Services is low, their use is difficult and appear consequently classified as very low by EGUI^+^ whereas in those where the quality of e-Services is high, they tend to be more used, and appear consequently classified as high by EGUI^+^ in general. For instance, Romania (RO) and Bulgaria (BG) remain in low positions in all the rankings with average differences with the four indexes compared of -2 and 2 positions respectively whereas Netherlands (NE) and Norway (NO) and remain in high positions in all the rankings with average rank differences of -1 and 2 positions in the four comparisons.

On the other hand, only 10.6% of the countries (blue-bold ones) on average showed higher positions in the rankings provided by the rest of the indexes than for that of EGUI^+^ (< − 5 positions), i.e. their theoretical situation seems to be better than the practical one. Analyzing all the groups with high negative ranking differences with EGUI^+^ (blue-bold ones), we did not find any country common to all of them but Belgium (BE) could be considered as common since it is nearly in the blue-bold group of the GU index. In addition, we observed that Belgium (BE) also was the country with higher drops, falling from 10^th^, 11^th^ and 10^rd^ positions in the EGDI, OSI and NRI rankings to the 17^th^ one in the EGUI^+^ ranking. Consequently, we could state that the real use of e-Government services is below the expectations we could have in Belgium.

Finally, 4.8% of the countries on average achieved lower positions (red-italic) in the rankings of the four conceptual indexes than in the ones provided by EGUI^+^ (>5 positions), although the EGDI one does not have any country with such ranking rises. In the rest of indexes, we found that there is not any common country to all the groups but Ireland (IE) could be considered as common as it is nearly in the red-italic group of NRI index. In addition, Ireland also was the country with the highest ranking rise for the index we defined, rising from 13^nd^ and 15^th^ positions in the OSI and GU rankings to 7^th^ one in that of EGUI^+^. This example would be in the opposite case of Belgium, the real use of e-Government services is above the expectations we could have in Ireland.

Beyond that, we observed greater differences between the values given by the index defined (EGUI^+^) for the different countries, specially when compared to EGDI and OSI (see columns 2-6 in Table 6). In this sense, although countries are similarly ranked they are more clearly distinguished according to the use of the e-Government services than according to the theoretical quality of such services. Those countries ranked in the first (Denmark) and last (Romania) positions according to EGUI^+^ are the clearest examples, with index values almost duplicating the values achieved by second best and worst ranked countries respectively.

For a deeper analysis of the similarity between the performance of the four conceptual indexes and that of EGUI^+^ we computed four pairwise comparisons based on Kendall correlation [23] using the rankings provided by each index. Table 7 shows the results of Kendall pairwise tests between EGUI^+^ and EGDI, OSI, NRI and GU, in terms correlation values (T) and significance (p-values). The second and third columns of the table show the performance of the stable countries (green-roman ones in Table 6) suggesting that the four indexes are highly correlated with EGUI^+^ at 0.05 significance level, p-value<alpha, with correlation values (T) on average of 0.8. In addition, in the in the fourth and fifth columns of Table 7 we also show the results of the Kendall tests carried out analyzing the complete set of countries. In this case, the values of T decreased down to 0.7 on average, being EGDI the index which scores the highest correlation value (T=0.72) with the index we defined. In the global comparison, we observed slightly higher correlation values for the EGDI index and its OSI index than for the NRI index and its GU pillar.

**Table 7:** Results of the Kendall pairwise correlation tests between EGUI^+^ and EGDI, OSI, NRI and GU.

	Stable* countries		26 countries	
Index	p-value	T	p-value	T
EGDI	8.90 × 10^−10^	0.78	8.14 × 10^−09^	0.72
OSI	1.09 × 10^−08^	0.79	2.00 × 10^−07^	0.67
NRI	3.05 × 10^−07^	0.75	2.00 × 10^−07^	0.67
GU	1.42 × 10^−08^	0.75	3.78 × 10^−07^	0.66

* Countries which showed small ranking differences (≤±5 positions) with EGUI^+^.

Considering all the above, we can state that the empirical analysis carried out on e-Government adoption across Europe through EGUI^+^ index concur to a large extent with the theoretical studies which measure the level of e-readiness of European countries through different indexes (EGDI, OSI, NRI and GU).

## Discussion

On the one hand the digital divide makes the task of providing universally accessible online government services challenging [12] and on the other hand, citizen confidence in the ability of an agency to provide online services is imperative for the widespread adoption of e-Government initiatives [14]. According to Shareef et al. [2], e-Government adoption behavior differs when functional characteristics of organizational, technological, economical, and social perspectives of e-Government differ. The first part of the empirical study carried out based on Eurostat CSIS Surveys, the classification of countries in different e-Government use levels (see Table 3) is in concordance with the statement since, first of all, not all countries have the same e-Government use level, and, although with exceptions, more developed and wealthier countries seem to have higher levels of e-Government use.

With regard to the factors affecting the e-Government use, buying goods in the Internet could be expected to be one of the factors directly related to e-Government use due to the similarities existing between e-commerce and e-Government. According to Schwester [12] the same way factors from Technology Acceptance, Diffusion of Innovation and trustworthiness models play a role in user acceptance of e-commerce, it is expected that they will also affect e-Government adoption. Accordingly, the outcome of our study shows that in countries with higher e-Government adoption according to Eursotat CSIS Surveys, IBUY, the variable related to e-commerce is most of the times correlated to the use or not use of e-Government services.

But, this is not always the case, there are differences between commercial businesses and government agencies [14]. E-commerce and e-Government differ in their reasons for existence (profit vs. service) and constituents served (target market vs. population at-large. Businesses can choose their customers; however, in e-Government, agencies are responsible for providing access to the entire eligible population, including individuals with lower incomes and disabilities [12]. Mandatory relationships exist only in e-Government. Citizens perceive businesses differently than government. In addition, the structure of businesses is different from the structure of agencies in the public sector. Decision-making authority is less centralized in government agencies than in businesses. This dispersion of authority impedes the development and implementation of new government services. The third difference is accountability. In a democratic government, public sector agencies are constrained by the requirement to allocate resources and provide services ‘in the best interest of the public’. The political nature of government agencies is also a feature that makes e-Government and e-commerce different. These factors could be related with the fact that in countries with lower adoption level (EGUI^+^ = low/very low), other factors such as education level and occupancy appear to be related to the e-Government adoption.

On the other hand, some authors as Afyonluoglu and Alkar [32] for instance, compared 16 international e-Government benchmarking studies completed between 2001—2016 by five active organizations including UN and WEF and identified the common points and the differences with respect to 22 different criteria including indexes such as EGDI and NRI. They pointed out that none of studies compared measures the “usage of e-services by citizens”, “governance model of e-Government”, “benefits of e-services” and “satisfaction”, suggesting that they should be considered for future e-Government framework improvements. Similarly, Jadi and Jie [33] use the EPI E-participating index, a supplementary indicator designed by the UN, as an output of government effort to evaluate the performance of e-Government systems. The authors state, that although the EGDI index is used as a benchmark to provide a numerical ranking of e-Government development, building websites, infrastructures and providing online services only shows how the readiness of the government to exploit the facilities is. However, in addition to those indicators the performance of e-Government systems can be analyzed by measuring to what extent citizens are using these facilities. The second part of this work is aligned with the previous lines since we compared the e-Government practical use and the e-readiness of 26 EU countries, based on the EGUI^+^ index, empirically computed from the 2009-2015 Eurostat CSIS Surveys, the EGDI index and its OSI component published in the 2010-2016 UN e-Government Surveys, and the NRI and its GU component provided by the 2010-2015 World Economic Forum’s Global Information Technology Reports. According to our analysis, it seems that in the majority of the countries the situation of the e-Government does not differ substantially despite using different calculation methods. To this regard we think that the compute of e-readiness of countries (EGDI, NRI) could be improved by including the real use of e-Services quantified in the index we define (EGUI^+^).

Finally, the outcome of the index comparisons points some countries where we could focus to analyze other types of variables influencing the quality and use of the services provided by the Governments. That is, the analysis of politics, infrastructures and other aspects in countries where the quality of the provided services and the e-Government services use level does not match, such as Belgium and Ireland, could probably give important clues about aspects affecting to the use of these services.

## Conclusions

In this paper we analyzed e-Government adoption, the practical use of e-Services supplied by Governments, across Europe (for 26 EU countries) based on the empirical data provided by Eurostat [13]. The outcomes contribute to gaining insight on some of the factors influencing the e-Government adoption in Europe and can provide some guidelines to improve the interaction of citizens with web services and information offered by institutions depending on their e-Government use level.

The data was first used to quantify the adoption level by defining two indexes: the E-Government Use Index (EGUI) and an extreme version of it, taking only into account the highest e-Government adoption for calculation (EGUI^+^). The countries were then rated according to four adoption levels based on EGUI^+^ levels (very high, high, low and very low) and two countries from each level were selected to characterize the e-Government adoption using supervised learning procedures, CTC trees [17]. In particular, the system was able to differentiate individuals doing null e-Goverment use from individual doing complete use of it with an average accuracy of 73% in the eight countries selected: Denmark and Norway, Ireland and Estonia, Latvia and Belgium and Poland and Romania. This enabled us to identify the main factors affecting the practical use of e-Government tools for eight different countries, which can contribute to better understand of these values. Furthermore, the main rules provided by the eight trees built, were supported by the Pearson correlation computed for the 26 EU countries.

Specifically, Pearson revealed that European citizens who had bought goods quite recently over the Internet did use e-Government tools and, although according to CTC the complete use of e-Government tools is also determined by other specific factors, the same conclusion arouse from the CTC structures of all the countries selected except for Romania. On the other hand, Pearson’s analysis determined that European citizens with high education levels do usually a complete use of e-Government tools what was also observed in the CTC trees of six of the eight countries analyzed with very high (Norway), high (Ireland and Estonia), low (Latvia and Belgium) and very low (Poland) EGUI^+^ levels, combined with other types of factors.

Finally, for the 26 EU countries analyzed, we compared the rankings of the EGUI^+^ index to that of other conceptual indexes measuring the level of e-readiness of a country such as the E-Government Development Index (EGDI) its Online Service Index (OSI) component, the Networked Readiness Index (NRI) and its Government usage component (GU). As a result, EGUI^+^ was found to be correlated with the four indexes mentioned at 0.05 significance level showing that adoption levels extracted from the empirical analysis are in general aligned with more conceptual and theoretical values.

In summary, we think that our research results provide some key-aspects that could be considered for future strategic decisions on the improvement of e-Government adoption in different European countries, in terms of knowledge about the most influential factors on null and complete e-Government use and also in terms of proposing complementary indexes based on empirical data.

With regard to future work our study could be extended to include new Eurostat (2016-2020) and UN (2018-2020) data and involve other e-Government indicators suggested by various authors [33–35]. On the other hand, a new analysis could be carried out excluding the countries with no Eurostat data for any of the years, e.g. Belgium (2015), Latvia (2009). Additionally, the research could be enriched by extending the geographical area of interest or focusing more closely on a smaller area. Finally, the analysis of other variables affecting the use and infrastructures of e-Services could help understanding the differences found regarding the countries achieving the best values for the main indexes used (EGUI^+^, EGDI and NRI).

## Supporting information

S1 Appendix (PDF)
