# Supplementary material for: Characterization of e-Government adoption in Europe
Source: PLoS One. 2020 Apr 17;15(4):e0231585. doi: 10.1371/journal.pone.0231585 (PMC7164598; doi:10.1371/journal.pone.0231585)
Supplement: S1 Appendix — (PDF) [file pone.0231585.s001.pdf]

Appendix 1

Table 8. Mode differences of the variables selected from the Eurostat CSIS Surveys in the period 2009-2015 for the 26 EU countries analyzed.

	Mode					
Country	HH_IQ	AGECLS	SEX	ISCED	IBUY	EGU
AT	4	5	1	2	3	1
BE	3	4	1	3	1	1
BG	4	4	1	2	1	1
CY	2	3	2	2	1	1
CZ	4	4	2	2	1	1
DK	4	5	2	2	3	4
EE	4	2	2	2	1	1
EL	2	4	2	2	1	1
ES	2	4	2	3	1	1
FI	4	5	2	2	3	4
FR	4	5	1	2	3	4
HU	4	4	2	2	1	1
IE	NA*	4	2	3	3	4
IT	4	4	1	2	1	1
LT	4	5	2	2	1	1
LU	4	5	1	3	3	1
LV	4	4	2	2	1	1
MT	4	3	1	1	3	1
NL	4	5	2	2	3	4
NO	4	5	1	2	3	4
PL	4	2	2	2	1	1
PT	4	4	2	1	1	1
RO	4	2	1	2	1	1
SE	4	4	1	2	3	4
SI	4	3	2	2	1	1
SK	3	3	2	2	1	1

* No data was collected.
